# Changes in Reproductive Health Information-Seeking Behaviors After the Dobbs Decision: Systematic Search of the Wikimedia Database

**DOI:** 10.2196/64577

**Published:** 2024-12-16

**Authors:** Mackenzie Lemieux, Cyrus Zhou, Caroline Cary, Jeannie Kelly

**Affiliations:** 1 Department of Obstetrics and Gynecology Washington University School of Medicine in St. Louis St Louis, MO United States

**Keywords:** abortion, Dobbs, internet, viewer trends, Wikipedia, women’s health, contraception, contraceptive, trend, information seeking, page view, reproductive, reproduction

## Abstract

**Background:**

After the US Supreme Court overturned *Roe v. Wade*, confusion followed regarding the legality of abortion in different states across the country. Recent studies found increased Google searches for abortion-related terms in restricted states after the *Dobbs* *v. Jackson Women’s Health Organization* decision was leaked. As patients and providers use Wikipedia (Wikimedia Foundation) as a predominant medical information source, we hypothesized that changes in reproductive health information-seeking behavior could be better understood by examining Wikipedia article traffic.

**Objective:**

This study aimed to examine trends in Wikipedia usage for abortion and contraception information before and after the *Dobbs* decision*.*

**Methods:**

Page views of abortion- and contraception-related Wikipedia pages were scraped. Temporal changes in page views before and after the *Dobbs* decision were then analyzed to explore changes in baseline views, differences in views for abortion-related information in states with restrictive abortion laws versus nonrestrictive states, and viewer trends on contraception-related pages.

**Results:**

Wikipedia articles related to abortion topics had significantly increased page views following the leaked and final *Dobbs* decision. There was a 103-fold increase in the page views for the Wikipedia article *Roe v. Wade* following the *Dobbs* decision leak (mean 372,654, SD 135,478 vs mean 3614, SD 248; *P<*.001) and a 67-fold increase in page views following the release of the final *Dobbs* decision (mean 8942, SD 402 vs mean 595,871, SD 178,649; *P*<.001). Articles about abortion in the most restrictive states had a greater increase in page views (mean 40.6, SD 12.7; 18/51, 35% states) than articles about abortion in states with some restrictions or protections (mean 26.8, SD 7.3; 24/51, 47% states; *P*<.001) and in the most protective states (mean 20.6, SD 5.7; 8/51, 16% states; *P*<.001). Finally, views to pages about common contraceptive methods significantly increased after the *Dobbs* decision. “Vasectomy” page views increased by 183% (*P*<.001), “IUD” (intrauterine device) page views increased by 80% (*P*<.001), “Combined oral contraceptive pill” page views increased by 24% (*P*<.001), “Emergency Contraception” page views increased by 224% (*P*<.001), and “Tubal ligation” page views increased by 92% (*P*<.001).

**Conclusions:**

People sought information on Wikipedia about abortion and contraception at increased rates after the *Dobbs* decision. Increased traffic to abortion-related Wikipedia articles correlated to the restrictiveness of state abortion policies. Increased interest in contraception-related pages reflects the increased demand for contraceptives observed after the *Dobbs* decision. Our work positions Wikipedia as an important source of reproductive health information and demands increased attention to maintain and improve Wikipedia as a reliable source of health information after the *Dobbs* decision.

## Introduction

In the United States, public interest in reproductive health information has been affected by recent drastic changes to the political landscape. In *Dobbs v Jackson Women’s Health Organization*, a landmark case decided on June 24, 2022, the Supreme Court overturned *Roe v. Wade*, ruling that the US Constitution does not protect the right to abortion and leaving the states to decide its legality [1,2]. Since June 2022, the legality and accessibility of abortion have changed rapidly and significantly across the country. As of August 2024, a total of 14 states have outlawed abortion with few exceptions, 4 states have enacted 6-week abortion bans [3], and 14 states have adopted or are working to adopt amendments to enshrine the right to abortion in their constitutions [4,5]. These changes have created considerable confusion among the public about the legality of abortion. One 2023 survey found that 45% of the public was unsure if medication abortion was legal in their state [6].

Recent papers published in *JAMA Health Forum* and *JAMA Internal Medicine* [7,8] have explored health information-seeking behaviors in the post-*Dobbs* era to understand the public health impact of increasingly strict legislation on reproductive health care. These studies revealed that Google searches for abortion, contraception, and reproductive health-related topics reached record levels after the *Dobbs* decision was leaked, especially in states with restrictive abortion policies. Importantly, the increased demand for reproductive health information is complicated by the overwhelming amount of abortion misinformation on the internet. Experts have warned that there is currently an abortion “infodemic,” [9] a term the World Health Organization (WHO) defines as the rapid spread of an excessive amount of health information, some of which is false, that ultimately confuses the public and decreases trust in the medical field [10]. Maintaining up-to-date online information about reproductive care is imperative for people to stay informed and understand how policy decisions impact their health.

While Google search trends [7,8] provide a surface-level view of internet user behavior, Wikipedia page views reflect an in-depth interest in a topic [11]. Compared with Google, studying Wikipedia also offers more specificity regarding where internet users obtain their health information. Furthermore, the process of crowdsourcing information on Wikipedia represents an opportunity for intervention in the dissemination of accurate reproductive and public health information, as attempted by the WHO during COVID-19 [12].

Research into who uses Wikipedia and why shows that 90% of medical students used Wikipedia to aid in their studies [13], 70% of junior physicians consulted Wikipedia in a clinical setting [14], and 35% of pharmacists reported using Wikipedia at work in a questionnaire [15]. In 2019, Wikipedia was ranked the second most commonly used resource for health information online [9], potentially due to its presence as a top-10 search result 71% to 85% of the time across common search engines such as Google, Yahoo, MSN, and so on [16]. During the COVID-19 pandemic, there was a significant increase in the number of medical articles on Wikipedia, a finding published in the *Journal of Medical Internet Research* in 2021 [17]. The authors of this article suggest that studying Wikipedia traffic could be a valid approach to epidemiologic surveillance [17]. Given the widespread use of Wikipedia as a source of health information for health professionals, students, and patients alike [18,19], we sought to explore viewing trends on Wikipedia pages about abortion policies, medical and surgical abortion, and contraceptive options after the *Dobbs* decision. We hypothesized that Wikipedia is used to obtain reproductive health information more after the *Dobbs* decision and more in restrictive states.

## Methods

### Data Scraping

A total of 89 Wikipedia articles were examined for this study. The process of choosing these articles and why they were chosen is further detailed below in the *Selection of Reproductive Health-Related Wikipedia Pages* section. A list of all 89 articles and their Wikipedia links can be found in Multimedia Appendix 1. Daily view counts to 87 total Wikipedia articles were obtained for 2 years bookending the June 24, 2022, *Dobbs* decision, from June 24, 2021, to June 24, 2023, using the *pageview* library (version 0.5.0) with the following options: platform “all” and user type “user.”

**Figure 1:**
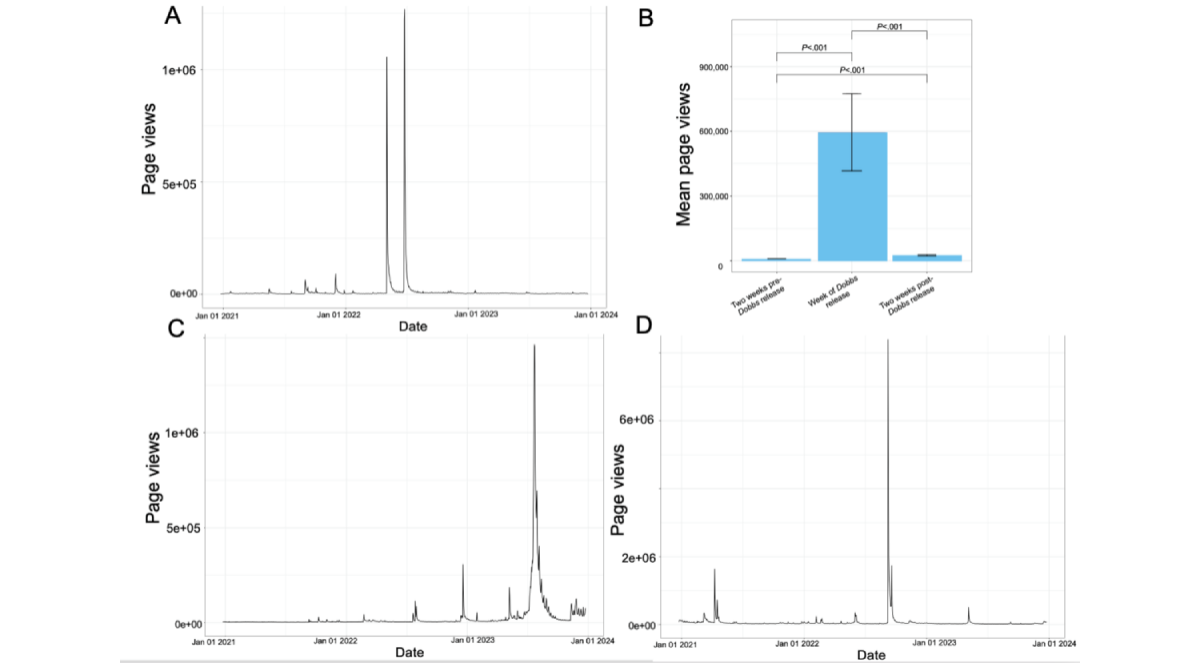
(A) A line graph of page view counts over time for the English-language Wikipedia page “Roe v. Wade” for 3 years bookending the Dobbs decision (between January 2021 and January 2024). (B) A bar chart comparing mean page views across a 2-week span before, 1-week span during, and 2-week span after the Dobbs decision was made in June 2022. (C) and (D) Line graphs depicting page view counts over the 3 years bookending the Dobbs decision for 2 control pages: “Elizabeth II” (Queen Elizabeth II) and “J. Robert Oppenheimer.”.

Longitudinal trends in page views of the Wikipedia article *Roe v. Wade* were assessed given the central role of this abortion-related political event to our research question. Longitudinal trends in page views to 2 prominent topics surrounding the *Dobbs* decision with associated Wikipedia articles were selected as controls: Queen Elizabeth II and J. Robert Oppenheimer. Daily view counts for the 3 years bookending the *Dobbs* decision were obtained for these 3 pages and plotted in a histogram format (Figure 1).

### Selection of Reproductive Health-Related Wikipedia Pages

Given that abortion policy varied dramatically by state following the *Dobbs* decision, we sought to determine if Wikipedia page viewing patterns differed by state. Using categories defined by The Guttmacher Institute [3], we grouped individual states into the following categories: restrictive policies, protective policies, and varied restrictive or protective policies. We subsequently quantified views to Wikipedia pages dedicated to each US state’s abortion laws (ie, “Abortion in Missouri” and “Abortion in Florida”). A list of the specific Wikipedia articles is provided in Table 1.

We next examined the percentage change in page views for 3 subsets of pages providing abortion information, including medical, surgical, and self-managed abortion. For medical abortion, we selected pages for the 2 most commonly used abortion medications, “Mifepristone” and “Misoprostol,” as described by the WHO and the American College of Obstetricians and Gynecologists (ACOG) [20,21]. Planned Parenthood (PPFA) and the Guttmacher Institute, 2 well-regarded sources of information within the field of obstetrics and gynecology, also recognize these 2 medications as the most commonly used for medical abortions [22,23]. We then selected blue hyperlinked alternative abortion medications found within the Wikipedia “Mifepristone” and “Misoprostol” articles to simulate Wikipedia user behavior. Similarly, the surgical abortion pages “Vacuum Aspiration” and “Dilation and Extraction” were selected based on PPFA’s web page for procedural abortion [24], and again, we selected blue hyperlinked alternative procedural abortion pages found within the “Vacuum Aspiration” and “Dilation and Extraction” pages. Finally, we examined the Wikipedia article for “Self-induced abortion” and selected the blue-linked alternative on this page, “Menstrual extraction.” A list of all included pages can be found in Table 2.

**Figure 2:**
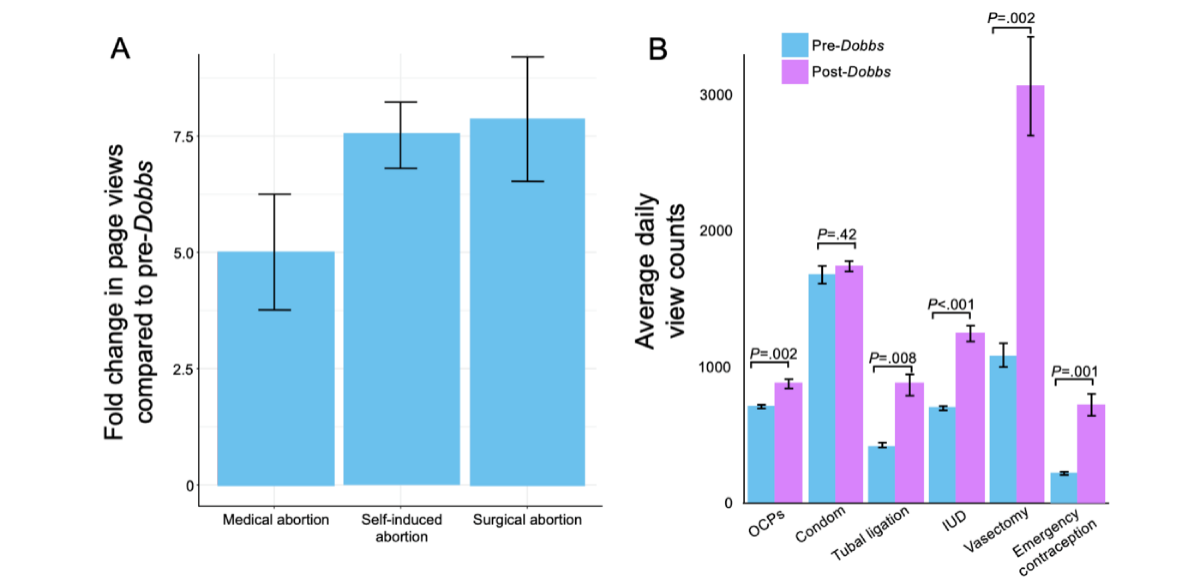
Bar charts depicting changes in page views for abortion- and contraception-related pages during the 2 weeks before the Dobbs decision and the week after the Dobbs decision. (A) The fold change in page views compared with the baseline for pages about medication abortions, self-induced abortions, and surgical abortion procedures. (B) Comparison of average daily page view counts before and after the Dobbs decision for the following pages, which are combined: oral contraceptives (OCPs), condoms, tubal ligation, intrauterine device (IUD), vasectomy, and emergency contraception.

We next examined percentage changes in page views compared with the baseline for pages about the most commonly used birth control methods as described in the Guttmacher Institute 2021 report [15], including “Vasectomy,” “Tubal ligation,” “IUD” (intrauterine device), “Combined oral contraceptive pill,” “Emergency Contraceptive,” and “Condoms” (Figure 2B).

Examining Wikipedia pages with titles that exhibit more “colloquial language” was a consideration; however, many Wikipedia search attempts using colloquial language “redirect” to one common page that encompasses the colloquial terms. For example, the following Wikipedia search terms redirect to the Wikipedia page “Emergency Contraception” instead of having their own dedicated page: “Morning-after-pill,” “Emergency Contraceptive Pill,” “Emergency Birth Control,” “Postcoital contraception,” “Next day pill,” “Plan B Contraceptive,” and so on. We similarly found many colloquial Wikipedia search terms that redirect to the pages “Medical Abortion,” “Dilation and Curettage,” “Contraceptive Implant,” “Birth Control,” and “Tubal Ligation,” to name a few. Thus, we felt that we were capturing searches in which daily speech or colloquial language was used, given that Wikipedia has streamlined these search terms to one common page in many instances.

**Table 1:** Change in page views for US state abortion pagesa.

Abortion protectiveness and state			Daily page views, mean (SD)			Fold change
			Before the *Dobbs* decision		After the *Dobbs* decision	
**Protective**						
	Minnesota	27 (7.4)		834 (590.2)		30.9
	Maryland	26 (6.6)		758 (529.9)		29.7
	Oregon	40 (11.6)		907 (553.4)		22.7
	New Jersey	49 (8.0)		963 (649.8)		19.8
	Vermont	13 (8.3)		250 (128.5)		19.7
	California	114 (31.8)		1929 (1216.7)		16.9
	New York	96 (16.4)		1312 (686.2)		13.6
	New Mexico	30 (8.4)		350 (277.4)		11.9
**Varied**						
	Ohio	46 (5.7)		2135 (1191.2)		46.6
	Utah	23 (4.5)		996 (730.1)		43.6
	Delaware	5 (2.4)		221 (162.9)		43.0
	Montana	8 (4.6)		325 (246)		43.0
	Arizona	29 (8.0)		1184 (446.2)		41.0
	Florida	70 (31.9)		2415 (1228.8)		34.3
	Wisconsin	37 (7.5)		1156 (1554.5)		30.9
	Colorado	83 (8.2)		2395 (1274.8)		28.9
	Alaska	22 (5.8)		634 (243.5)		28.6
	Connecticut	16 (5.5)		433 (229)		26.6
	Nevada	17 (3.7)		436 (342.1)		25.4
	Michigan	30 (6)		755 (433.4)		25.3
	New Hampshire	19 (7.2)		429 (473.2)		23.1
	Pennsylvania	35 (4)		796 (647.3)		22.9
	Wyoming	11 (4.8)		244 (206.3)		21.9
	Kansas	47 (8.9)		959 (275.5)		20.6
	Illinois	60 (16.7)		1222 (716.2)		20.5
	Hawaii	16 (3.8)		323 (202.2)		20.4
	Iowa	14 (5.2)		288 (165.4)		20.4
	Rhode Island	10 (2)		188 (185.1)		18.8
	Massachusetts	30 (7.8)		541 (357.7)		17.8
	Maine	16 (8.1)		262 (150)		16.1
	Washington	23 (5)		302 (430.6)		13.1
	Virginia	33 (4.4)		356 (496.6)		10.9
**Restrictive**						
	Kentucky	13 (3.2)		1142 (641.6)		85.9
	Missouri	40 (6.3)		3204 (2149.2)		79.5
	Idaho	14 (5.1)		846 (581.1)		60.4
	Alabama	51 (7.7)		2976 (1581.7)		58.0
	North Dakota	8 (4.1)		440 (208.1)		55.0
	Arkansas	20 (5.5)		1043 (557.1)		52.2
	Mississippi	27 (9.9)		1163 (887.3)		42.4
	Tennessee	42 (9.4)		1691 (1252.3)		39.9
	Nebraska	9 (2.9)		344 (310.8)		38.2
	Louisiana	32 (15.8)		1140 (654.1)		36.1
	Georgia	44 (9.2)		1469 (935.9)		33.3
	Indiana	25 (6.2)		729 (402.1)		29.1
	North Carolina	39 (6.4)		1023 (1037.1)		26.1
	Texas	270 (36.6)		6081 (4503.4)		22.6
	South Dakota	21 (14.2)		470 (305.2)		22.4
	West Virginia	13 (4.2)		224 (498.1)		17.6
	South Carolina	26 (6.2)		441 (545.5)		17.2
	Oklahoma	108 (16.4)		1581 (1012.3)		14.7

^a^Mean page views and fold change in page views before and after the *Dobbs* decision for each US state’s abortion policy Wikipedia page. Within each restrictiveness category, states are ordered from highest to lowest fold change.

**Table 2:** Changes in page views for abortion-related pages before and after the Dobbs decisiona.

Articles and their categories			Before the *Dobbs* decision, mean (SD)		After the *Dobbs* decision, mean (SD)		Fold change		*P* value
**Medical**									
	Abortifacient	278 (70.9)		2347 (1250.6)		8.5		.01	
	Gemeprost	10 (3.4)		17.4 (5.3)		1.7		.01	
	Medical abortion	299 (121.8)		2149 (1306.7)		7.2		.01	
	Methotrexate	1140 (149.2)		1640 (260.4)		1.4		.001	
	Mifepristone	491 (54.7)		3575 (1265.9)		7.3		<.001	
	Misoprostol	749 (69.9)		2953 (1299.7)		3.9		.004	
**Surgical**									
	Dilation and curettage	403 (35.0)		2013 (705.0)		5.0		<.001	
	Dilation and evacuation	169 (45.7)		1144 (379.8)		6.8		<.001	
	Hysterotomy abortion	30 (6.8)		255 (90.5)		8.5		<.001	
	Instillation abortion	37 (7.1)		305 (113.2)		8.2		<.001	
	Intact dilation and extraction	136 (32.1)		1872 (655.0)		13.8		<.001	
	Vacuum aspiration	146 (28.2)		726 (240.6)		8.2		.01	
**Self-induced**									
	Menstrual extraction	64 (11.7)		530 (314.2)		6.8		.004	
	Self-induced abortion	346 (38.4)		2353 (1152.7)		5.0		<.001	

^a^Mean page views and fold change in page views before and after the *Dobbs* decision for all abortion-related pages included in the subcategory analysis of medical, self-induced, and surgical abortion.

### Data Cleaning and Statistics

Data were transformed and summarized with the aid of the *dplyr* (v 1.1.3) and *scales* (v 1.2.1; both developed in R Studios) libraries. Data visualization and graphics were generated using *ggplot2* (v 3.4.4) and *ggpubr* (v 0.6.0). All statistic comparisons were performed using Wilcoxon rank-sum (Mann-Whitney *U* test). In cases where multiple comparisons were made, *P* values were adjusted using false discovery rate correction, which was performed using the Benjamini-Hochberg procedure. All data analysis was done using R (R Core Team) (version 4.1.2) using the R studio interface (PositPBC; v 2023.06.2).

### Ethical Considerations

This study does not include human subjects research (no human subjects experimentation or intervention was conducted) and so does not require institutional review board approval.

## Results

### The Public Seeks Information About Current Events on Wikipedia, Including Abortion Legislation

During the study period, 2 prominent spikes in page views were observed for the Wikipedia article *Roe v.* *Wade* (Figure 1A)*.* These 2 spikes corresponded to the first week of May (when the *Dobbs* decision was leaked to the public on May 2, 2022) and the fourth week of June (when the *Dobbs* decision was formally released).

A comparison of the average daily page views to the Wikipedia article *Roe v.* *Wade* 2 weeks before the *Dobbs* leak (April 17 to May 1, 2022) and the week of the *Dobbs* leak (May 2–9, 2022) revealed a significant 103-fold increase (mean 372,654, SD 135,478 vs mean 3614, SD 248; *P<*.001) in the number of views after the leak. Similarly, when comparing the average daily page views 2 weeks before the *Dobbs* decision (June 9-23, 2022) to the week of the *Dobbs* decision (June 24 to July 1, 2022), we found a significant 67-fold increase in the number of views the week the *Dobbs* decision was released (Figure 1B; mean 8942, SD 402 vs mean 595,871, SD 178,649; *P*<.001). This pre- versus post-*Dobbs* spike in view counts was not seen in selected Wikipedia control articles for prominent media or news events unrelated to abortion. Wikipedia articles for J. Robert Oppenheimer (Figure 1C) and Queen Elizabeth II (Figure 1D), which saw spikes in other parts of the year, did not have observable spikes in early May and late June as the *Roe v. Wade* article did (Figure 1C and D).

### State Abortion Restrictions Impact Information-Seeking Behavior on Wikipedia

**Figure 3:**
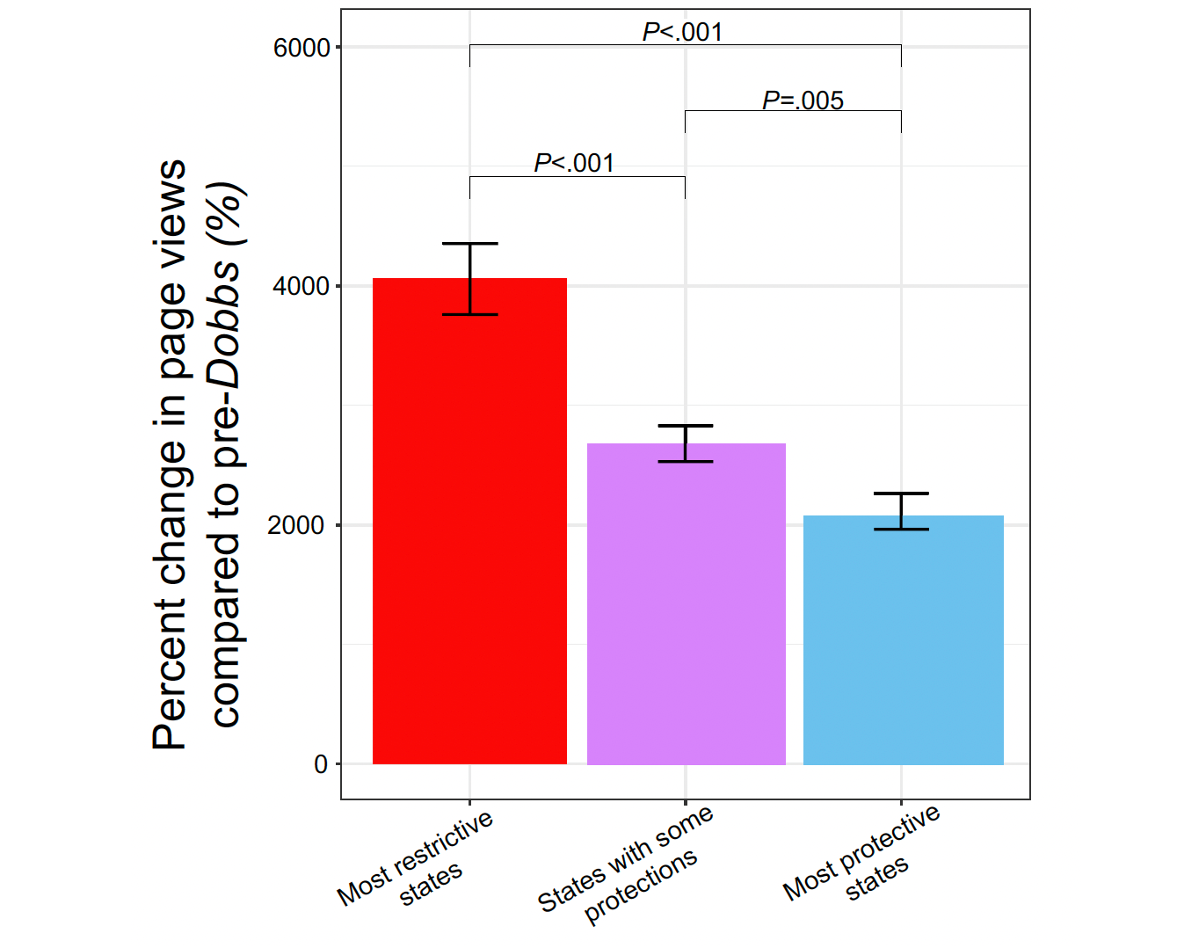
Bar chart comparing percentage change in page views before and after the Dobbs decision for US state abortion pages (eg, “Abortion in Texas” and “Abortion in California”) on Wikipedia. States were stratified based on abortion restriction status guided by Guttmacher Institute data.

Across all states, we observed a significant increase in searches for Wikipedia articles covering state-level abortion policies following the *Dobbs* decision. Similar to what we observed with the view counts in the *Roe v. Wade* article, 2 prominent spikes in viewership correlated to the week of the *Dobbs* leak and the *Dobbs* decision. In aggregate, we saw a roughly 30-fold increase in views for state-level Wikipedia articles following the *Dobbs* decision (eg, the average daily views of the Wikipedia article “Abortion in Alaska” went from 22 views before the *Dobbs* decision to 634 views after the *Dobbs* decision). Although all states had a significant increase following the *Dobbs* release, the relative magnitude of the increase significantly varied by the restrictiveness of state abortion laws (Figure 3 and Table 1). The percentage change in page views was significantly higher for most or very restrictive states (mean 40.6, SD 12.7; 18/51, 35% states) compared with states with some restrictions or protections (mean 26.8, SD 7.3; 24/51, 47% states; *P*<.001) and to states with most protections (mean 20.6, SD 5.7; 8/51, 16% states; *P*<.001).

### Abortion and Contraceptive Information-Seeking Behavior Increased After the Dobbs Decision on Wikipedia

Views to Wikipedia pages about medical, surgical, and self-managed abortion significantly increased during the week of the *Dobbs* decision compared with baseline (2-week average before the decision), corresponding to a 401% (SD 124%), 687% (SD 72%), and 652% (SD 133%) change, respectively (Figure 2A). The individual pages comprising each subcategory of abortion information are found in Table 2.

Compared with the baseline, views for the page “Vasectomy” increased by 183% after the *Dobbs* decision (*P*<.001), views for the page “IUD” increased by 79% after the *Dobbs* decision (*P*<.001), and views for the page “Combined oral contraceptive pill” increased by 24% after the *Dobbs* decision (*P*<.001). Views for the page “Tubal ligation” increased significantly by 92% after the *Dobbs* decision compared with baseline (*P*=.01), as did views for the page “Emergency Contraception” (*P*<.001), which increased by 224%. However, views for the page “Condoms” did not increase significantly after the *Dobbs* decision compared with baseline (*P*=.42; Figure 2B).

## Discussion

### Principal Findings

Our study provides unique insight into reproductive health information-seeking behavior surrounding the landmark *Dobbs* decision. To our knowledge, this is the first study using Wikipedia as a tool to examine reproductive health information-seeking behaviors on the internet. Through scraping page views of abortion and contraception-related Wikipedia pages, we found increased viewer traffic to these pages surrounding the leak and the final *Dobbs* decision. Public interest in the *Roe v. Wade* Wikipedia page correlated with current events and public interest in state abortion laws related to states’ hostility toward abortion. Finally, public interest in common abortion and contraception methods was significantly higher after the *Dobbs* decision. Our findings situate Wikipedia as a source of reproductive health information in English and highlight the need to maintain Wikipedia as an information resource.

### Public Use of Wikipedia

The breadth of knowledge on Wikipedia is unparalleled with over 59 million articles in over 300 languages [25]. Of these pages, 30,000 are about health and medicine topics in English and another 164,000 are about health and medicine topics in other languages [26]. Given the extent of medical information on Wikipedia, it is unsurprising that Wikipedia has been used to explore health behavior trends. Wikipedia page view trends have been used to estimate peak weeks of influenza-like illness ahead of the Centers for Disease Control and Prevention with 17% more accuracy than Google trends [27]. Wikipedia page view trends have also been used to monitor and forecast disease-location pairs across the globe [28]. More recently, a study published in the *Journal of Medical Internet Research* in 2021 showed that there was a significant increase in medical article submissions during the height of the COVID-19 pandemic [17]. The authors further postulate that observing Wikipedia activity could be a viable method of epidemiologic surveillance [17]. However, no research to date has explored health behavior trends on Wikipedia concerning reproductive health.

### Dynamic Abortion Policies and Misinformation

The current state of abortion misinformation has been widely regarded as harmful to the health, rights, and freedoms of people living in the United States [9]. Since the *Dobbs* decision, Americans have been increasingly pressed to find trustworthy information on the internet about abortion rights and policies in their communities [9,29,30]. The right to free and reliable medical information is documented and described by the WHO [31], yet this is not the reality for most individuals.

Interestingly, during the COVID-19 pandemic, when misinformation was rampant, the WHO partnered with Wikipedia to address misinformation [12]. By immediately updating Wikipedia pages with new information from the WHO, the partnership was an attempt to disseminate new and reputable information quickly and to as many people as possible [12]. Given our findings, it is reasonable to consider a future partnership between Wikipedia and institutions such as ACOG, PPFA, and the Guttmacher Institute to disseminate accurate and up-to-date information surrounding reproductive health, especially in light of the upcoming election in November where reproductive rights are at stake.

One of our main findings is that we see larger increases in views for pages about abortion in hostile US states, which mirrors previous research showing an increase in internet searches about abortion in states with the most severe restrictions by up to 42% after the *Dobbs* decision [7,8]. One possible interpretation of this finding is that an increase in abortion information-seeking behavior in hostile states reflects a lack of knowledge or understanding surrounding the legality of abortion in restrictive states. Post-*Dobbs* increases in depression and anxiety symptoms among residents of abortion-hostile states, particularly among women of reproductive age [32,33], also support this speculation. Increased levels of misinformation [34] and dynamic discussions about the future of abortion rights in hostile states have the potential to further contribute to increased searches about abortion information. Interestingly, the “restrictive” states with the highest fold change in views, Kentucky and Missouri, already have or are planning to place abortion rights on state ballots [35]; the “varied” state with the highest fold change, Ohio, has enshrined abortion rights in their state constitution through a ballot measure already [36].

### Contraceptive Trends After the Dobbs Decision

In addition to Wikipedia page views, internet searches for contraception increased after the *Dobbs* decision [37], particularly for permanent methods like vasectomy and tubal ligation [7,38]. According to PPFA, traffic to their web page on sterilization procedures increased by 300% in the first month after the *Dobbs* decision [39]. Increased internet searches corresponded to increased demand for these methods. At PPFA, birth control appointments increased by 15%, and appointments specifically for IUDs increased by 30% over the first month after the *Dobbs* decision [39]. Some institutions documented significant increases in female sterilization after the *Dobbs* decision*,* and patients cited *Dobbs* as an influence on their decision [40]. A study at one large US health care organization found a 160% increase in vasectomy procedures within 6 months after the *Dobbs* decision [41]. One company that provides contraception through mail saw a 30% increase in dual orders of emergency contraception and birth control pills after the *Dobbs* decision [37]. Heightened uncertainty about the entire reproductive health care landscape in the US could contribute to increased information-seeking and demand for the most effective contraceptive methods [37]. We did not find a significant increase in views for the “Condoms” page despite their frequent use [42] and evidence that condom use increased after the *Dobbs* decision [43]. Potential reasons for this include the accessibility of condoms compared with other forms of contraception, the lack of scrutiny toward condoms compared with other birth control methods [44], and potentially an elevated baseline knowledge of this method compared with other birth control methods.

### Wikipedia and the Right to Health Information

Our study shows that the public seeks reproductive health information on Wikipedia, and at significantly higher rates in times of political change. Given that Wikipedia is free and available to anybody with internet access, we argue that Wikipedia is an important provider of free reproductive health information. Promoting Wikipedia editing within the medical and health care communities is an important step toward maintaining and improving the amount of reliable, evidence-based reproductive health information on the internet. More than half of Wikipedia’s main editors have a health care background, and over 85% have a university education [45]. Many editors of health care–related pages are a part of WikiProject Medicine, whose recent focus has been incorporating Wikipedia editing into medical school curricula [46]. Especially in the current political climate of misinformation, it is imperative to maintain and improve reproductive health articles on Wikipedia. As mentioned above, one way we hypothesize to disseminate accurate and up-to-date health information is through partnerships between Wikipedia and large reproductive health organizations such as ACOG and PPFA [12].

### Limitations

Our study was limited to analyzing the behaviors of people with internet access and topics specifically searched within Wikipedia, not the entire internet. Our study was also limited to English-language Wikipedia and we cannot make conclusions about abortion information-seeking behavior for non–English-language speakers. While many users of English-language Wikipedia are located in the United States, our study is not limited to only US Wikipedia users. Given these limitations, our conclusions are specific to understanding if and how Wikipedia is used as a reproductive health information source in isolation, not in comparison to other internet information sources. While we cannot conclude anything about reproductive health information–seeking behavior on other internet platforms at this time, we do feel confident that Wikipedia is a source of reproductive health information for users seeking information in English. Our conclusions can be extended and acted upon in the context of Wikipedia alone. For example, we suggest that reproductive health organizations partner with Wikipedia to further expand on and disseminate reproductive health information. Future research could examine how often users consult reproductive health information on Wikipedia versus other online platforms and could be extended to users in languages other than English. There is potential for non–English-language users to consult different sources than English users.

### Conclusions

Our research contributes to the growing subset of public health literature that analyzes trends in reproductive health information-seeking behavior on the internet. With a goal of improving access to free and reliable reproductive health information, our work highlights the role Wikipedia plays in providing reproductive health information surrounding changes to reproductive health policy. Medical schools and professional organizations can support activities geared toward improving and creating reproductive health pages on Wikipedia and partner with Wikipedia to disseminate accurate and up-to-date reproductive health information.

## Appendix

Multimedia Appendix 1 List of the links to all Wikipedia articles analyzed for the purposes of this paper.

## Abbreviations

ACOG: American College of Obstetricians and Gynecologists
IUD: intrauterine device
PPFA: Planned Parenthood
WHO: World Health Organization
